# Utilization Patterns of Glucagon-Like Peptide-1 Receptor Agonists in Patients with Type 2 Diabetes Mellitus in Italy: A Retrospective Cohort Study

**DOI:** 10.1007/s13300-018-0396-2

**Published:** 2018-03-10

**Authors:** Marco Orsini Federici, Janette McQuillan, Giovanni Biricolti, Serena Losi, Jeremie Lebrec, Catrina Richards, Cristiana Miglio, Kirsi Norrbacka

**Affiliations:** 1 Eli Lilly SPA, Via A. Gramsci, 731-733, 50019 Sesto Fiorentino, FI Italy; 2 QuintileIMS, 210 Pentonville Road, London, N19JY UK; 3 Eli Lilly SpA, Via Thailandia, 27, 00144 Rome, Italy; 4 Eli Lilly Deutschland GmbH, Werner-Reimers-Straße 2-4, 61352 Bad Homburg, Germany; 5 Eli Lilly Finland, Laajalahdentie 23, 00330 Helsinki, Finland

**Keywords:** Glucagon-like peptide-1 receptor agonists (GLP-1 RAs), Persistence, Prescribed average daily dose, Treatment modifications, Type two diabetes

## Abstract

**Introduction:**

Real-world evidence on glucagon-like peptide-1 receptor agonist (GLP-1 RAs) usage is emerging in different European countries but is lacking in Italy. This retrospective cohort study aimed to describe the real-world drug utilization patterns in patients initiating GLP-1 RAs for treating T2DM in Italy.

**Methods:**

Adults aged ≥ 20 years and with ≥ 1 oral antidiabetic drug (alone or in combination with insulin) other than GLP-1 RAs in the 6 months prior to initiating exenatide twice daily (exBID), exenatide once weekly (exQW), dulaglutide once weekly (DULA), liraglutide once daily (LIRA) or lixisenatide once daily (LIXI) between March and July 2016 were retrospectively identified in the Italian IMS LifeLink™ longitudinal prescriptions database (retail pharmacy data). Patients with ≥ 6-month follow-up (defined as evidence of any prescription activity) were included. Proportions of patients who remained persistent (continued treatment until discontinuation/switch) in the first 6 months and of those who discontinued or switched to a different GLP-1 RA over the entire follow-up were recorded. For each treatment, the average daily/weekly dosage (ADD/AWD) while persistent during the available follow-up was calculated.

**Results:**

We identified 7319 patients: 92 exBID, 970 exQW, 3368 DULA, 2573 LIRA and 316 LIXI. Across treatments, 89% patients were ≥ 50 years old, 54% were males, and the median follow-up duration ranged between 8.1 and 8.7 months. At 6 months, 35% exBID, 47% exQW, 62% DULA, 50% LIRA and 40% LIXI patients remained persistent. Over the entire follow-up, median persistence days varied from 73 (exBID) to > 300 days (DULA). The mean ± SD ADD/AWD was exBID: 17.7 ± 2.1 µg/day; exQW: 2.1 ± 0.1 mg/week; DULA: 1.5 ± 0.2 mg/week; LIRA: 1.5 ± 0.2 mg/day; LIXI: 21.0 ± 5.5 µg/day.

**Conclusions:**

This real-world analysis suggests differences exist in persistence between patients treated with various GLP-1 RAs. Among the investigated treatments, patients prescribed exBID recorded the lowest and those prescribed DULA the highest persistence with therapy.

**Funding:**

Eli Lilly and Co., Indianapolis, IN, USA.

**Electronic supplementary material:**

The online version of this article (10.1007/s13300-018-0396-2) contains supplementary material, which is available to authorized users.

## Introduction

In Italy, 3.5 million people have been diagnosed with diabetes, over 90% of whom have type 2 diabetes mellitus (T2DM), with an estimated burden for the Italian national health system of approximately €8–10 billion yearly for drugs, hospitalizations and visits [1, 2].

Despite recent advances that have expanded the available therapeutic options, achieving and maintaining glycemic control in patients with T2DM remains a major challenge for doctors who must face complex therapeutic decisions [3]. The 2016 Italian Standard of Care [4], in line with the updated 2015 joint position statement released by the American Diabetes Association and European Association for the Study of Diabetes [5], recommends a patient-centered approach with metformin as the first line of therapy and the addition of one (or two) of the other available treatments as the second (or third) line of therapy, with no indicated preference. Doctors should make their initial decision based on the patient's physical and clinical characteristics and the efficacy and safety profiles of the available treatments and then address the eventual progressive worsening of glycemic control through treatment intensification.

Glucagon like peptide-1 receptor agonists (GLP-1 RAs) are a relatively new class of injectable drugs that have emerged as an attractive second- or third-line therapeutic option because of their association with improved glycemic control, lower hypoglycemia rate and weight loss, although gastrointestinal GLP-1 RAs side effects have also been reported [6, 7]. There are several GLP-1 RAs on the market in Europe, each with specific characteristics [3]; they vary in the magnitude of their effect in reducing HbA1c and enhancing weight loss and also in their adverse event profiles [3, 8]. Additionally, injection frequencies are variable, with some GLP-1 RAs offering more convenient dosing (weekly vs. daily injections) and potential higher adherence [9] compared with others. In Italy, the currently available GLP-1 RAs include exenatide twice daily (from now on referred to as exBID), launched February 2008; liraglutide once daily (LIRA), launched August 2010; exenatide once weekly (exQW) and lixisenatide once daily (LIXI), both launched December 2013 and dulaglutide (DULA) once weekly launched February 2016.

For a drug to be effective, patients must adhere to and persist with therapy. Previous studies have shown an association between better adherence and persistence with glucose-lowering treatments and improved clinical and economic diabetes-related outcomes [10–13]. Persistence with GLP-1 RAs has also been reported to be associated with positive clinical outcomes and reduced healthcare costs [14, 15]. However, to date, real-world data on persistence, discontinuation or switch dynamics with GLP-1 RA are limited. Only a few studies have compared treatment patterns or variable dosing across the different treatments [9, 15–20], and none of them were conducted in Italy.

Given the current limited evidence and the important clinical and costs implications, the main objective of this study was to provide real-world evidence on treatment patterns for T2DM GLP-1 RA therapy initiators in Italy, specifically persistence with the GLP-1 RA (i.e., index therapy) and treatment modifications, including discontinuation, switch to another hyperglycemic therapy, dosing changes and augmentation (addition of new therapy to the index treatment). Other objectives included evaluating the average daily/weekly dose (ADD/AWD) of the index therapy and describing the baseline characteristics of the patients initiating the different GLP1-RA treatments. These outcomes were retrospectively evaluated using the Italian Longitudinal Prescription (LRx) database, a large database containing information on retail dispensing.

## Methods

This study was a retrospective cohort study, including all patients receiving the first prescription of GLP-1 RA as exBID, exQW, DULA, LIRA or LIXI. To allow for comparisons, the overall study design and methodologies were like those used in previous studies conducted in other European countries [16, 17]. Ethics committee approval is not required for secondary use of pseudonymized prescription data in Italy.

### Data Source

The Italian LRx database (QuintileIMS, Durham, NC, and Danbury, CT, USA) accesses nationwide pharmacy data centers processing prescription data of all products reimbursed by the National Health System (class “A” products) or those reimbursed under specific circumstances, as per guidelines issued by the Italian Medicines Agency (class “A_PHT” products). Pharmacies are required to report details of these prescriptions to the Italian Ministry of Health to receive reimbursement. LRx covers 90% of prescriptions in the retail channel (weighted percentage between 90% and 73%) in Italy. Drugs dispensed in the hospital are not captured within LRx. However, hospital dispensation is expected to account only for a minority (< 10%) of the overall GLP-1 RA units sold in Italy (IQVIA MIDAS^®^ data; not shown).

Data are entered at the point of sale from retail pharmacies based on prescriptions that have been dispensed. Data are then uploaded from sites to a vendor where they are collated and further pseudonymized (de-identified).

Patient and prescription details in LRx include: sex and 5-year age band and prescription data, including EphMRA Anatomical Classification (ATC) code, quantity dispensed, prescriber specialty and date of dispensation. Diagnoses are not recorded in LRx.

### Study Design and Population Selection

**Fig. 1:**
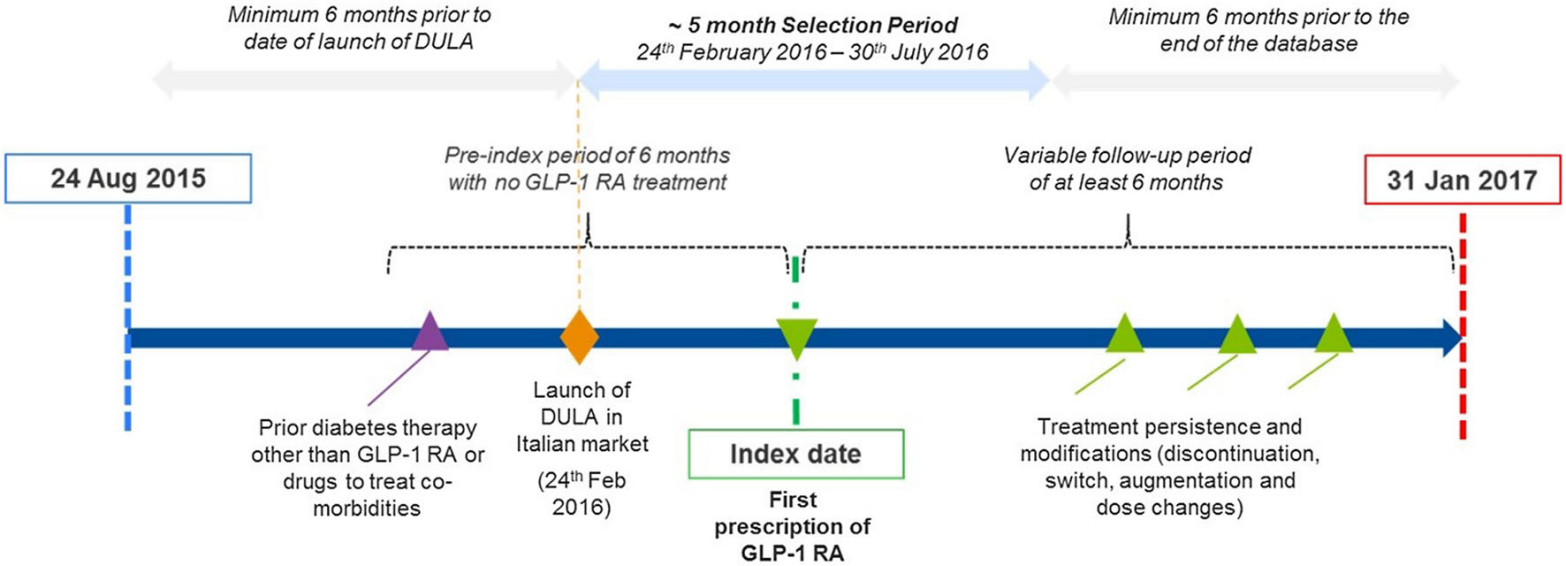
Overview of study design. *DULA* dulaglutide, *GLP*-*1 RA* glucagon-like peptide-1 receptor agonist

An overview of the study design is provided in Fig. 1. The study period was from 24 August 2015 (6 months prior to the most recently launched GLP-1 RA in Italy, DULA) to 31 January 2017 (last available data at the time of analyses). The index date was the date of first prescription for any of the GLP-1 RAs of interest (exBID, exQW, DULA, LIRA or LIXI) and the initiated therapy was termed the index treatment. Patients were included in the study if they had a 6-month continuous eligibility period (based on evidence of any prescription activity within the database) prior to and after the index date for baseline characterization and outcome evaluation. Therefore, the eligible index date was between 24 February 2016 and 30 July 2016. Patients were required to have received at least one prescription for other antidiabetic drugs, including oral treatments alone or in combination with insulins (proxy for T2DM diagnosis) and no GLP-1 RA treatment in the 6 months prior to the index date (look-back period). All the available follow-up from the index date until the earliest of the end of study period or end of continuous eligibility period were used for outcome evaluation, resulting in variable follow-up durations with a minimum of 6 months for each patient.

Only adult patients were analyzed in line with the GLP-1 RA indication. As age is only recorded in the database in 5-year bands, patients aged ≥ 20 years at the start of GLP-1 therapy were selected. Once selected, patients were assigned to one of the five study cohorts, based on the index treatment received: exBID, exQW, DULA, LIRA or LIXI cohorts. Patients prescribed liraglutide indicated for weight management (Saxenda^®^) were excluded from the analyses.

### Measures and Analyses

Baseline characteristics were described for all patients and by treatment cohort and included age band at the time of GLP-1 RA treatment initiation, gender and record of any antihyperglycemic therapy class other than GLP-1 RA (and the number of classes used) or other drugs to treat comorbidities prescribed in the 6 months prior to the index date. Other antihyperglycemic therapy classes of interest were oral anti-diabetics or insulins, including alpha glucosidase inhibitors, biguanide (i.e., metformin), dipeptidyl peptidase (DPP)-4 inhibitors, meglitinides, sodium-glucose co-transporter (SGLT)-2 inhibitors, sulfonylurea, thiazolidinediones, fixed oral combinations, short-acting insulins, basal insulins and pre-mixed insulins. Treatments for comorbidities included antidepressants, anti-emetics, weight-loss medication (including anti-obesity medications apart from Saxenda), anti-platelet medication, cardiovascular (CV) medication, antihypertensive drugs, anti-arrhythmic drugs, lipid-lowering agents and non-steroidal anti-inflammatory drugs (NSAIDs).

For all patients and by study cohort, the mean and standard deviation (SD) duration of follow-up in days was calculated. The resulting duration was divided by 30 days to obtain the follow-up duration in months (irrespective of calendar months). Over this time, the proportions and percentage of patients who experienced treatment modifications, including discontinuation, switch, dose changes and augmentation of therapy were evaluated. In addition, persistence was evaluated at the fixed 6-month minimum follow-up in terms of persistence proportions (and 95% CI) and over the variable follow-up period in terms of probability of remaining persistent over time and median (and 95% CI) persistence duration (days), using the Kaplan-Meier method.

Patients were considered persistent (i.e., on continued index therapy) until evidence of discontinuation or switch. Discontinuation was defined as the occurrence of a gap in a series of successive prescriptions that was > 2 times the expected drug lasting time as per prescription. The day after the expected drug lasting time as per prescription end was defined as the date of discontinuation. A repeat prescription of the patients’ index medication after this gap period was considered a restart and was not classified as continuation of the index therapy. A prescription of a new non-index therapy (including other GLP-1 RAs) within 30 days prior to or following a discontinuation was considered a change of therapy (switch). The date of the new non-index prescription was defined as the date of switch. Treatment augmentation was defined as a new non-index antihyperglycemic prescription (other than GLP-1 RAs), started over 30 days prior to the end of follow-up or the index discontinuation date. Dose changes included down- or up-titration, defined as any dose decrease or increase of the index therapy. If the dose increase was within the recommended licensed dose as indicated in the summaries of product characteristics [21], then the dose change was defined as on-label up-titration; if the dose increase was beyond the licensed doses, then it was defined as off-label up-titration.

The ADD of the index therapy was assessed for all patients over the available follow-up duration while on continued index treatment (i.e., while persistent), irrespective of any dose change or augmentation of therapy. Daily dose was calculated by dividing the total amount or units of drug prescribed by the number of days between two consecutive prescriptions. ADD was initially evaluated by calendar month intervals for patients with an index therapy prescription within that month. Average ADDs over calendar months were summarized to provide an overall ADD. An average weekly dose (AWD) was calculated for exQW and DULA by multiplying the daily dose by 7. More details on the calculation of ADD can be found in Divino et al. [17].

All analyses performed in this study were descriptive, and no statistical tests were performed to formally compare the treatment cohorts. This article does not contain any new studies with human or animal subjects performed by any of the authors.

## Results

### Selected Population and Baseline Characteristics

The population attrition is shown in Figure S1 in the supplementary material.

A total of 7319 patients met the inclusion criteria and were analyzed for their patient characteristics (Table 1), treatment utilization (Table 2) and average daily doses (Table 3). The majority were males (54%), and 89% were aged 50 years or older. Of the selected population, 1% (*N* = 92) of patients initiated treatment with exBID, 13% (*N* = 970) with exQW, 46% (*N* = 3368) with DULA, 35% (*N* = 2573) with LIRA and 4% (*N* = 316) with LIXI. While the age distribution was similar across the different treatment cohorts, there was a higher proportion of females who started treatment with exBID (60%) compared with other GLP-1 RA therapies (44–50%).

**Table 1:** Baseline patient characteristics

	All study GLP-1 RAs	exBID	exQW	DULA	LIRA	LIXI
Patients, *n* (%)	7319 (100)	92 (1.3)	970 (13.3)	3368 (46.0)	2573 (35.2)	316 (4.3)
Age at index year, *n* (%)						
20–29	17 (0.2)	0 (0.0)	2 (0.2)	8 (0.2)	7 (0.3)	0 (0.0)
30–39	120 (1.6)	0 (0.0)	15 (1.6)	61 (1.8)	36 (1.4)	8 (2.5)
40–49	682 (9.3)	7 (7.6)	99 (10.2)	320 (9.5)	224 (8.7)	32 (10.1)
50–64	3344 (45.7)	34 (37.0)	466 (48.0)	1536 (45.6)	1168 (45.4)	140 (44.3)
≥ 65	3156 (43.1)	51 (55.4)	388 (40.0)	1443 (42.8)	1138 (44.2)	136 (43.0)
Gender, *n* (%)						
Female	3338 (45.6)	55 (59.8)	432 (44.5)	1474 (43.8)	1218 (47.3)	159 (50.3)
Male	3981 (54.4)	37 (40.2)	538 (55.5)	1894 (56.2)	1355 (52.7)	157 (49.7)
Prior antidiabetic therapy other than GLP-1 RA, *n* (%)^a^						
None	156 (2.1)	3 (3.2)	34 (3.5)	52 (1.5)	65 (2.5)	2 (0.6)
AGI	364 (4.9)	4 (4.3)	45 (4.6)	148 (4.3)	149 (5.7)	18 (5.6)
Metformin	5713 (78.0)	77 (83.6)	761 (78.4)	2556 (75.8)	2056 (79.9)	263 (83.2)
DPP-4 inhibitors	2010 (27.4)	4 (4.3)	242 (24.9)	1205 (35.7)	489 (19.0)	70 (22.1)
Fixed oral combinations	2228 (30.4)	9 (9.7)	273 (28.1)	1273 (37.7)	602 (23.3)	71 (22.4)
Meglitinides	645 (8.8)	10 (10.8)	80 (8.2)	279 (8.2)	238 (9.2)	38 (12.0)
Basal insulins	1354 (18.4)	13 (14.1)	98 (10.1)	382 (11.3)	734 (28.5)	127 (40.1)
Short-acting insulins	603 (8.2)	4 (4.3)	50 (5.1)	172 (5.1)	325 (12.6)	52 (16.4)
Pre-mix insulins	44 (0.6)	0 (0.0)	6 (0.6)	15 (0.4)	18 (0.6)	5 (1.5)
SGLT-2 inhibitors	397 (5.4)	5 (5.4)	53 (5.4)	213 (6.3)	117 (4.5)	9 (2.8)
Sulfonylurea	2213 (30.2)	25 (27.1)	306 (31.5)	1100 (32.6)	684 (26.5)	98 (31.0)
Glitazones	835 (11.4)	6 (6.5)	120 (12.3)	440 (13.0)	240 (9.3)	29 (9.1)
Number of prior antidiabetic therapy classes used (other than GLP-1 RAs), *n* (%)^a,b^						
0	156 (2.1)	3 (3.2)	34 (3.5)	52 (1.5)	65 (2.5)	2 (0.6)
1	2626 (35.8)	46 (50)	381 (39.2)	1155 (34.2)	950 (36.9)	94 (29.7)
2	2573 (35.1)	31 (33.6)	344 (35.4)	1330 (39.4)	785 (30.5)	83 (26.2)
3+	1964 (26.8)	12 (13.0)	211 (21.8)	831 (24.7)	773 (30.0)	137 (43.4)
Other prior therapy, *n* (%)^b^						
Antidepressants	857 (11.7)	15 (16.3)	115 (11.8)	349 (10.3)	343 (13.3)	35 (11.0)
Anti-emetics	8 (0.1)	0 (0.0)	0 (0.0)	2 (0.0)	5 (0.1)	1 (0.3)
Weight-loss medication	0 (0.0)	0 (0.0)	0 (0.0)	0 (0.0)	0 (0.0)	0 (0.0)
Anti-platelet medication	2493 (34.0)	36 (39.1)	332 (34.2)	1062 (31.5)	939 (36.4)	124 (39.2)
CVD medication	6321 (86.3)	83 (90.2)	823 (84.8)	2877 (85.4)	2261 (87.8)	277 (87.6)
Antihypertensives	5683 (77.6)	78 (84.7)	731 (75.3)	2553 (75.8)	2060 (80.0)	261 (82.5)
Antiarrhythmics	154 (2.1)	2 (2.1)	18 (1.8)	56 (1.6)	68 (2.6)	10 (3.1)
Lipid-lowering drugs	3977 (54.3)	57 (61.9)	502 (51.7)	1814 (53.8)	1423 (55.3)	181 (57.2)
NSAIDs	1911 (26.1)	26 (28.2)	270 (27.8)	869 (25.8)	666 (25.8)	80 (25.3)

*exBID* exenatide twice daily, *exQW* exenatide once weekly, *DULA* dulaglutide, *LIRA* liraglutide, *LIXI* lixisenatide. *AGI* alpha glucosidase inhibitors, *DPP* dipeptidyl peptidase, *SGLT* sodium-glucose co-transporter, *CVD* cardiovascular disease, *NSAIDs* non-steroidal anti-inflammatory drugs

^a^Patients on fixed combinations were considered to be on two different drug classes

^b^Prescribed in the 6 months prior to the index date (i.e., start of GLP-1 RA treatment)

**Table 2:** Persistence with the index therapy and treatment modifications over the available follow-up duration

	All study GLP-1 RAs	exBID	exQW	DULA^a^	LIRA	LIXI
Patients, *n* (%)	7319 (100)	92 (1.3)	970 (13.3)	3368 (46)	2573 (35.2)	316 (4.3)
Persistence, i.e., no discontinuation or switch of the index treatment						
At 6 months, *N* (%)	3984 (54.4)	32 (34.8)	451 (46.5)	2084 (61.9)	1291 (50.2)	126 (39.9)
95% CI of proportions	53.3, 55.6	25.1, 44.5	43.4, 49.6	60.2, 63.5	48.2, 52.1	34.5, 45.3
Persistence duration (days between the index date and end of follow-up)						
Median (95% CI)	238 (219, 293)	73 (62, 140)	150 (128, 176)	> 300^a^	183 (167, 203)	113 (88, 151)
25th percentile	62	62	58	86	63	51
At least a first index treatment modification (including dose changes)						
*N* (%)	5073 (69.3)	73 (79.3)	610 (62.9)	2231 (66.2)	1886 (73.3)	273 (86.4)
First treatment modification type, *N* (%)^b^						
On-label up-titration	305 (4.2)	4 (4.3)	0 (0.0)	161 (4.8)	140 (5.4)	0 (0.0)
Off-label up-titration	178 (2.4)	1 (1.1)	0 (0.0)	89 (2.6)	57 (2.2)	31 (9.8)
Down-titration	791 (10.8)	2 (2.2)	0 (0.0)	531 (15.8)	214 (8.3)	44 (13.9)
Augmentation	437 (6.0)	5 (5.4)	57 (5.9)	197 (5.8)	162 (6.3)	16 (5.1)
Discontinuation	2956 (40.4)	55 (59.8)	497 (51.2)	1086 (32.2)	1164 (45.2)	154 (48.7)
Switch	406 (5.5)	6 (6.5)	56 (5.8)	167 (5.0)	149 (5.8)	28 (8.9)
Up-titration at any time by type, *N* (%)^c^						
On-label up-titration	340 (4.6)	5 (5.4)	0 (0.0)	178 (5.3)	157 (6.1)	0 (0.0)
Off-label up-titration	247 (3.4)	1 (1.1)	0 (0.0)	126 (3.7)	81 (3.1)	39 (12.3)

*exBID* exenatide twice daily, *exQW* exenatide once weekly, *DULA* dulaglutide, *LIRA* liraglutide, *LIXI* lixisenatide

^a^Median persistence days for this cohort could not be calculated as more than 50% of the patients, over the entire follow-up, remained persistent

^b^Percentages are calculated over the total number of patients, overall and within each treatment cohorts

^c^Up-titration at any time was defined as any two consecutive prescriptions exceeding the index dose

**Table 3:** Overall average daily and weekly doses by treatment cohort

	exBID	exQW	DULA	LIRA	LIXI
Number of patients included in the analyses^a^	48	687	2742	1854	204
Average daily dose while persistent	mcg	mg	mg	mg	mcg
Mean (SD)	17.66 (2.11)	0.30 (0.02)	0.22 (0.03)	1.54 (0.22)	21.01 (5.45)
Median	16.55	0.30	0.22	1.49	19.34
Average weekly dose while persistent		mg	mg		
Mean (SD)	–	2.12 (0.14)	1.52 (0.19)	–	–
Median	–	2.08	1.52	–	–

*exBID* exenatide twice daily, *exQW* exenatide once weekly, *DULA* dulaglutide, *LIRA* liraglutide, *LIXI* lixisenatide, *SD* standard deviation (estimated neglecting repeated use of some patients), *mg* milligrams, *mcg* micrograms

^a^Average doses were calculated for patients who remained persistent with the index therapy and had at least two consecutive prescriptions for the index therapy

Overall, 78% patients were on metformin therapy in the 6 months prior to initiating GLP-1 RA treatment, with similar proportions across the different treatment cohorts (76–84%); 36% were on monotherapy (30–50% across the different cohorts), 35% on dual therapy (26–39% across the different cohorts), and 27% used three or more therapy classes (13–43% across the different cohorts). The most commonly used other non-antidiabetic treatments were for cardiovascular diseases (86% overall, 85–90% across different treatment cohorts), antihypertensive drugs (78%, 75–85%) and lipid-lowering medication (54%, 52–62%).

### Treatment Persistence and Modifications

The overall mean (SD) follow-up duration was similar across the different cohorts and was 8.3 (1.4) months, minimum 6.0 and maximum 11.3 months.

Although descriptive analysis was done, the results suggest that treatment patterns differed between treatment groups.

**Fig. 2:**
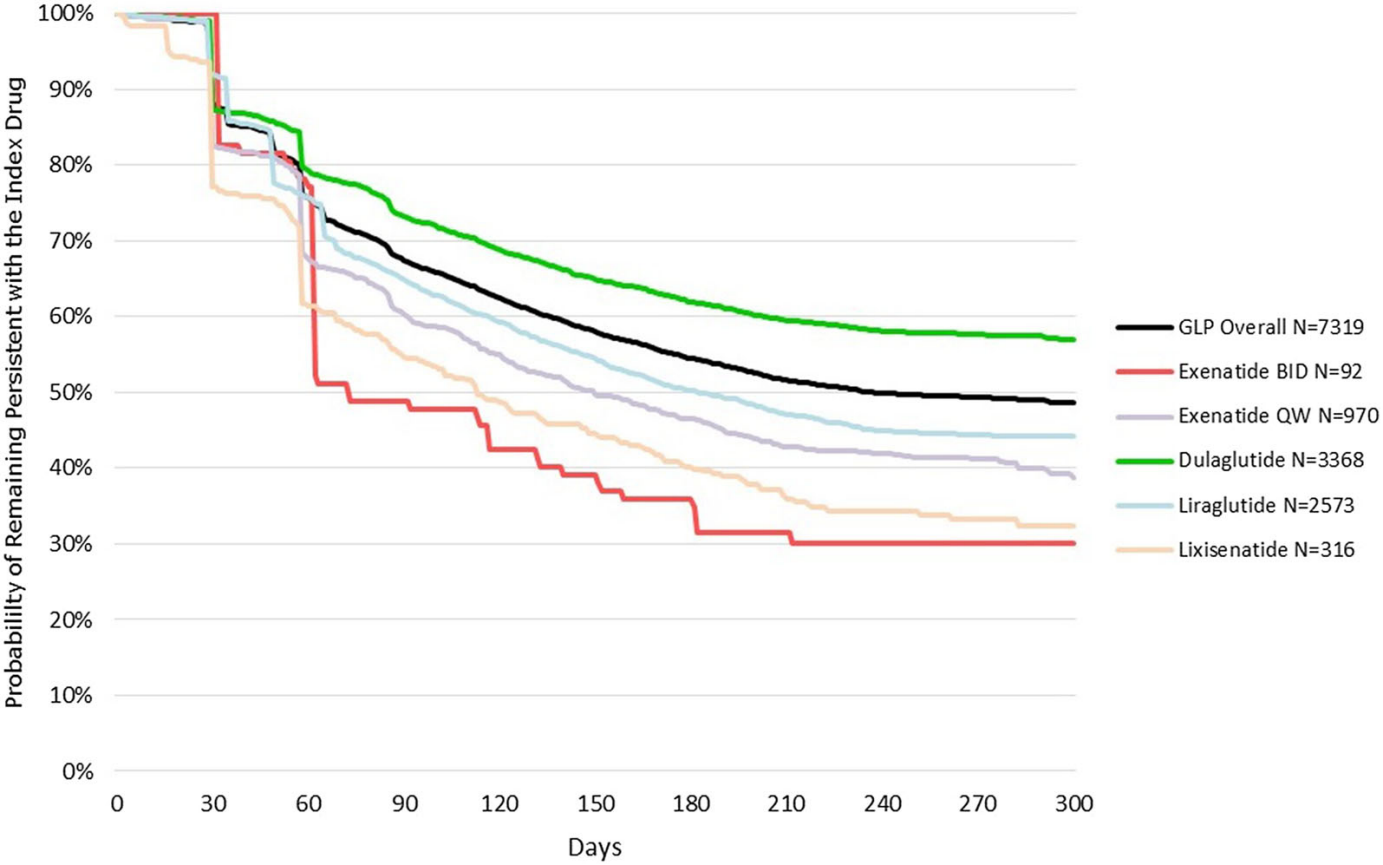
Kaplan-Meier analyses over the available follow-up durations: probability of remaining persistent with the index therapy. *exBID* exenatide twice daily, *exQW* exenatide once weekly, *DULA* dulaglutide, *LIRA* liraglutide, *LIXI* lixisenatide

Treatment persistence at 6 months, duration of persistence and treatment modifications over the available follow-up are shown in Table 2. Across all treatments, almost half of the patients on any GLP-1 RA remained persistent with their initial therapy (i.e., they did not discontinue or change the index treatment) at 6 months (54%; 95% CI 53, 56). This proportion was higher for patients on DULA (62%; 95% CI 60, 64) and lower for those on exBID (35%; 95% CI 25, 45) and LIXI (40%; 95% CI 35, 45) compared with exQW (47%; 95% CI 43, 50) and LIRA (50%; 48, 52). Over the full follow-up period, median persistence time (95% CI) varied from 73 days (62, 140) for patients on exBID to 183 days (167, 203) for patients on LIRA, while the median was not reached for patients on DULA (i.e., median time to end of persistence > 300 days) (Fig. 2). The fact that patients starting treatment with DULA had the highest probability of remaining persistent with the index therapy compared with other GLP-1 RAs at and beyond 6 months is illustrated by the Kaplan-Meier curves (Fig. 2). The highest risk of discontinuing or switching therapy was recorded in the first 2 months of treatment (Fig. 2).

The occurrence of treatment modifications also varied among the different treatment cohorts (Table 2). Over the available follow-up, 63% of patients on exQW and 66% of those on DULA experienced at least one treatment change compared with 79% on exBID and 73% on LIRA. The highest proportion of patients with at least one treatment modification was recorded for patients on LIXI (86%).

The exBID cohort recorded the highest proportion of patients whose first treatment change was a discontinuation (60%), while those on DULA recorded the lowest (32%). Patients on exQW did not experience dose changes as first treatment modification. Across other treatments, 5–10% of patients experienced a dose increase as a first treatment change, while 7–12% experienced a dose increase at any time. Switch (5–9%) and augmentation (5–6%) proportions were similar across the different treatment cohorts.

### Average Daily/Weekly Doses

According to the summary of product characteristics of the different GLP-1 RAs [22–24], the recommended initial dose of exBID is 5 µg twice daily, increased to 10 µg twice daily after 1 month; LIRA should be initiated with a dose of 0.6 mg once per day, which can be increased to 1.2 mg after at least a week. Doses can be further increased to 1.8 mg after at least another week on 1.2 mg for non-responsive patients; LIXI is also administered once per day, with a starting dose in the 2 weeks following initiation of 10 µg and with a fixed maintenance dose of 20 µg on day 15 and thereafter; exQW (2 mg) and DULA (0.75 mg as monotherapy or 1.5 mg in combination with other drugs) are administered once per week. The overall average daily and weekly dose over the available follow-up period recorded in this study is shown in Table 3 for all patients and by treatment cohort. For all treatments but LIXI, the prescribed ADD/AWD was in the ranges of the licensed doses. For LIXI, a slightly higher ADD of 21.0 µg was recorded.

## Discussion

The results of this retrospective analysis of Italian prescribing data show that there are differences in the real-world utilization patterns of different GLP-1 RAs. Previous real-world studies in Italy have examined diabetes-related outcomes related to GLP-1 RAs [25–27]; this is the first published study evaluating the real-world treatment patterns of individual GLP-1 RAs in Italy. The results add to the evidence base coming from other European countries as the number of GLP-1 RAs available on the European market increases.

Based on our findings, patients initiating treatment with DULA are more likely to remain persistent compared with other drugs, while greater proportions of patients initiating on exBID and LIXI tend to discontinue or switch treatment. Across the different treatments, persistence with the index therapy at 6 months was exBID < LIXI < exQW < LIRA < DULA. The KM analysis over the full follow-up period confirmed this trend and showed that patients initiating on DULA are less likely to discontinue or switch therapy than patients on other treatments. There are many possible reasons for low treatment persistence, including high injection frequency (twice daily vs. once daily vs. weekly injections), patient low tolerability of treatment, adverse reactions or lack of effectiveness. On the other hand, a higher persistence with therapy has been reported to result in better glycemic control and economic outcomes, with an estimated reduced healthcare cost of 27–30% [14, 15]. Although not ascertained as part of this study, therapies with improved persistence could be associated with better outcomes and lower costs.

The differences in treatment patterns observed in this study are consistent with those recorded in other European countries and in the US. In a similar analysis conducted in T2DM initiating GLP-1 RA treatment in 2013, Divino and colleagues [16] examined treatment patterns with exBID, exQW, LIRA and LIXI over 1 year from initiation in five European countries. The proportion of persistent patients was lower for exBID compared with LIRA and exQW in Germany (29% vs. 43% and 33%, respectively), The Netherlands (34% vs. 61% and 51%) and Sweden (31% vs. 59% and 43%). The proportion of persistent patients was also lower for exBID in Belgium compared with LIRA and LIXI (18% vs. 29% and 50%) and in France compared with LIRA (44% vs. 52%). Previously, the same authors [17] reported that persistence was lower for patients newly initiating on exBID (47–74% across Germany, Belgium, The Netherlands, UK and Sweden) compared with LIRA (51–80%) and exQW (58–75%) in the first 6 months of treatment. Alatorre and colleagues [9] looked at treatment patterns of patients initiating GLP-1 RA treatment with DULA vs. exQW and LIRA between November 2014 and April 2015 in the US. At 6 months from initiation, the proportion of patients discontinuing treatment was lower for DULA vs. exQW (26% vs. 48%) and for DULA versus LIRA (28% vs. 36%). While these trends are overall in line with the results of this study, different proportions across countries can be explained by the inherent characteristics of the local health systems as well as differences in the study designs, time when the studies were carried out and follow-up durations.

This study also found that the average daily doses prescribed to patients on the different GLP-1 RA treatments were generally within the recommended ranges, but for LIXI this was slightly above the recommended range (21.0 mg recorded in this study, label range 5–10 mg twice daily), suggesting that some patients on this drug may require higher doses to maintain or achieve glycemic control.

Differences in the methods used to calculate ADD may limit comparisons with other studies. The methodology used in this study was similar to that used by Divino and colleagues [16], who recorded an ADD of 1.55 mg for patients on LIRA in Germany, similar to this study. ADD varied in other countries and was lower in Belgium (1.41 mg) and higher in France (1.64 mg), The Netherlands (1.68 mg) and Sweden (1.60 mg). In the same study, the authors also recorded an ADD of 20.1 mg for patients on LIXI in Germany [16], in line with the current study, and confirm that slightly higher doses than expected for this treatment may be prescribed by doctors in the real world.

The authors acknowledge some limitations to the study design. First of all, we were unable to investigate reasons for treatment modifications (lack of effectiveness, adverse events, etc.) as these clinical details are not covered by prescription databases. Second, selection bias toward more severe patients may have occurred because of our continuous enrollment requirements based on prescribing activity. However, this effect should be minor in patients affected by chronic diseases, such as diabetes, who are regularly taking treatments over long period of times. In addition, the continuous enrollment criterion was necessary to ensure adequate visibility into the patients’ clinical history. Third, some of the GLP-1 RAs have been launched in Italy only recently; therefore, the average follow-up duration was short (< 1 year). Additional research is needed to confirm our results over longer time periods and to support clinically relevant findings with statistical testing. Finally, irrespective of the launch date of the different products in Italy, the sample size varied greatly across the different treatment cohorts, with only 92 patients identified who initiated treatment with exBID. This small sample size calls for caution in the interpretation of the exBID results.

Limitations consistent with a pharmacy-based database should also be taken into account when interpreting the results of this study. Patients who purchase prescriptions outside the pharmacies included in the database will not have that prescription utilization recorded in the database, which may result in an underestimation of the drug usage. Doses were calculated according to the dispensed packs over time, which means that stockpiling of prescriptions or filling prior to prescription run-out could lead to overestimations in dose. Furthermore, data are only collected from pharmacies that participate in the database, which may cause some levels of selection bias. However, the impact of this is considered to be very limited in the Italian LRx, as the database coverage is around 90% on average across all regions. Finally, pharmacy data do not record patient mortality. Consequently, patients may be considered discontinued because of no evidence of prescriptions after the minimum 6-month follow-up period rather than dead.

## Conclusions

This study adds to the limited literature on type 2 diabetes mellitus and GLP-1 receptor agonists by providing new evidence on the real-world usage of exenatide (twice daily and once weekly), dulaglutide, liraglutide and lixisenatide in Italy. Despite country-specific differences in databases, prescribing and dispensing practices and the analytical methods used, the results of the current analysis are aligned with those from other countries and indicate that patients on exenatide twice daily are less likely to remain persistent with their therapy than those on other GLP-1 RA treatments with either weekly (exenatide QW and dulaglutide) or daily dosing schedules (liraglutide and lixisenatide). Patients with the highest persistence initiated dulaglutide. In addition, for lixisenatide, the average daily dispensed doses as derived from pharmacy data may exceed the recommended doses indicated in the summary of product characteristics.

Given the importance of treatment persistence in maintaining glycemic control over time and reducing healthcare costs, these results suggest that dulaglutide may help improve clinical and economic outcomes for patients with T2DM initiating GLP-1 RA treatment. Further studies would be useful to explore the potential clinical and economic benefits associated within the GLP-1 RA class.

## Electronic supplementary material

Below is the link to the electronic supplementary material.

Figure S1. Selection of the study population. Abbreviations: *exBID* exenatide twice daily, *exQW* exenatide once weekly, *DULA* dulaglutide, *LIRA* liraglutide, *LIXI* lixisenatide (JPEG 188 kb)
