# Supplementary figures and images for: Utilization Patterns of Glucagon-Like Peptide-1 Receptor Agonists in Patients with Type 2 Diabetes Mellitus in Italy: A Retrospective Cohort Study
Source: Diabetes Ther. 2018 Mar 10;9(2):789–801. doi: 10.1007/s13300-018-0396-2 (PMC6104260; doi:10.1007/s13300-018-0396-2)

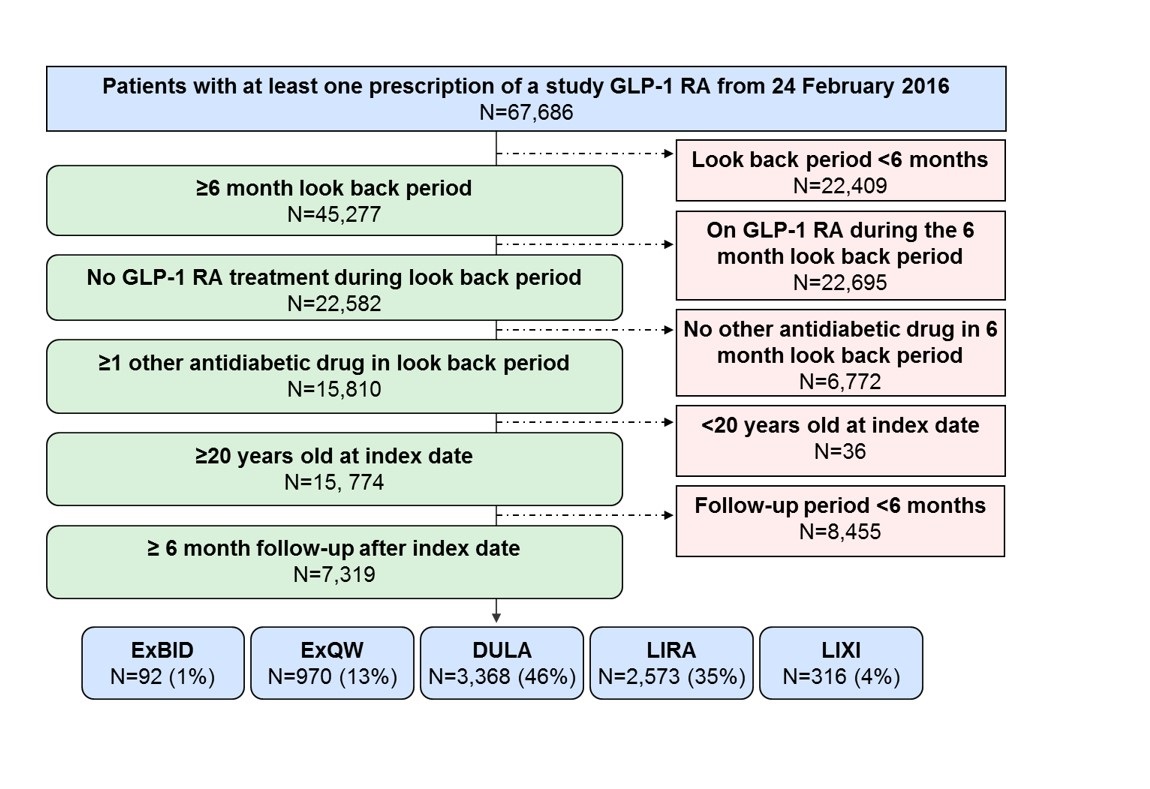

Supplement: Supplementary file 1 — Figure S1. Selection of the study population. Abbreviations: exBID exenatide twice daily, exQW exenatide once weekly, DULA dulaglutide, LIRA liraglutide, LIXI lixisenatide (JPEG 188 kb) [file 13300_2018_396_MOESM1_ESM.jpg]
